# Intratubular Biomineralization in a Root Canal Filled with Calcium-Enriched Material over 8 Years

**DOI:** 10.3390/ma10121388

**Published:** 2017-12-05

**Authors:** Yeon-Jee Yoo, Yoo Sang Lee, Jun Sang Yoo, Hiran Perinpanayagam, Chang Seon Yoo, Hyen Sug Kang, Soram Oh, Seok Woo Chang, Kee-Yeon Kum

**Affiliations:** 1 Department of Conservative Dentistry, Dental Research Institute, Seoul National University School of Dentistry, Daehakro 101, Seoul 03080, Korea; duswl0808@hanmail.net; 2 Department of Dentistry, Seoul National University School of Dentistry, Seoul 03080, Korea; troki@naver.com (Y.S.L.); dryjskr@daum.net (J.S.Y.); csyoo0712@naver.com (C.S.Y.); archedia@naver.com (H.S.K.); 3 Department of Dentistry, Schulich School of Medicine & Dentistry, University of Western Ontario, London, ON N6A 3K7, Canada; hperinpa@uwo.ca; 4 Department of Conservative Dentistry, School of Dentistry, Kyung Hee University, Seoul 02447, Korea; soram0123@naver.com (S.O.); swc2007smc@gmail.com (S.W.C.)

**Keywords:** biomineralization, calcium-enriched material, calcium-deficient hydroxyapatite, dentinal tubule, energy dispersive spectroscopy, scanning electron microscopy

## Abstract

This case report describes intratubular biomineralization in root canal, filled with calcium-enriched material after 8 years of clinical maintenance. The schematic findings of dentinal tubules were investigated with scanning electron microscopy (SEM) and energy dispersive spectroscopy (EDS). The root canal obturation material was closely adapted to root dentin surface, suggesting the possibility of chemical bonding between the two interfaces. SEM and EDS observation of dentinal tubules showed intratubular biomineralized crystal structures with Ca/P ratio in a range of 1.30–2.12, suggesting bioactive capacity of calcium-enriched material.

## 1. Introduction

For the root canal treatment of teeth, calcium-enriched materials such as mineral trioxide aggregate (MTA) have been proposed as an alternative to conventional gutta percha fillings. MTA is highly biocompatible, and provides an excellent seal, although it requires an extended setting time and is irretrievable for retreatments [1,2]. Regarding its performance in root-end fillings and perforation repairs, controversies exist with respect to its efficacy in providing an apical seal in orthograde fillings. MTA’s sealing effects appear to be due to its bioactive ability, forming hydroxyapatite crystals on material surfaces and inside the dentinal tubules, as reported by in vitro studies [3,4,5,6,7,8]. However, there is no compelling evidence on the long-term perspectives for the biomineralization in dentin, and its relevance is unclear. 

This case report provides the first long-term perspective of the biomineralization of dentin in root canal-treated teeth. Intratubular biomineralization within the dentinal tubules of a human permanent mandibular canine was observed more than 8 years after an orthograde obturation of the canal with MTA.

## 2. Clinical Presentation and Treatment

**Figure 1:**
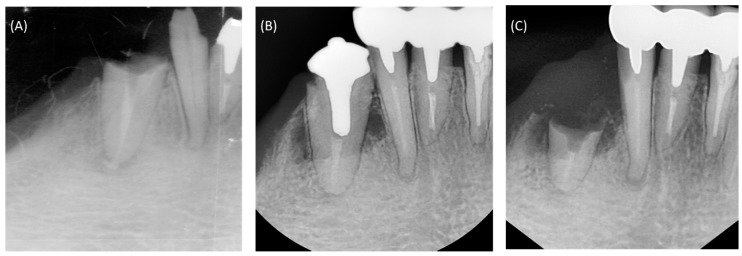
Intraoral periapical radiographs of the patient’s mandibular right anterior dentition. (**A**) At initial presentation, the canine had a subgingival fracture and prior root canal treatment. (**B**) The root canal was retreated and obturated with calcium-enriched material, and the tooth was restored with a gold cast post. (**C**) After 8 years, there was a subgingival root fracture below the level of crestal bone.

A 43-year-old woman presented to the dental clinic with the chief complaint of a fractured right mandibular canine. Clinical and radiographic examination revealed a subgingival fracture of the tooth, and prior treatment of the root canal that was associated with a periapical radiolucency (Figure 1A). A treatment plan was formulated for retreatment of the canal and restoration of the tooth, and informed consent was obtained from the patient. 

Local anesthesia was applied, rubber dam isolation was obtained, and the previous gutta percha filling material was removed from the root canal. Then, the canal was prepared with NiTi rotary files (ProTaper Universal F4, Dentsply Maillefer, Ballaigues, Switzerland), and the apical area was enlarged with manual stainless steel K files up to #45. During instrumentation, the canal was irrigated copiously with 5.25% sodium hypochlorite (NaOCl). Then, the canal was dried with paper points, filled with calcium hydroxide paste as an intracanal medicament, and the access was sealed with temporary filling material. After 2 weeks, the root canal was copiously irrigated with 5.25% NaOCl, then immersed in 17% ethylenediaminetetraacetic acid (EDTA) solution (pH 7.2) for 1 min, and finally flushed with 5.25% NaOCl, with all irrigants activated ultrasonically (P5 Newtron1 XS; Satelec, Acteon group, Mérignac, France). Then, the canal was copiously rinsed with sterile distilled water, dried with sterile paper points, and obturated with OrthoMTA (BioMTA, Seoul, Korea), as previously reported [8,9]. The OrthoMTA was mixed with distilled water by using the OrthoMTA automixer (BioMTA), as recommended by the manufacturer. The paste was introduced into the canal with the OrthoMTA carrier (BioMTA), and applied to the canal wall using OrthoMTA compactor, which has a 25/0.02 tip. The compactor was inserted to working length and rotated with a circumferential filing motion at 60 rpm. Sterile dry ISO-standardized paper points (Meta Dental Corp., Chungbuk, Korea) that were of the size of the apical preparation were used to compact the MTA cement at the root canal apex. After obtaining an apical plug, additional paper points of increasing size according the taper of the canal were used to further compact MTA up to 7 mm from the apex. After one week, the patient was recalled to confirm that the MTA cement had adequately set, and the tooth was prepared for a cast post restoration. The tooth was finally restored with gold cast post (Figure 1B), and full veneer gold crown was placed as an abutment of removable denture in lower arch. 

The patient was recalled at regular at 6-month intervals for the careful examination and evaluation of the root canal treatment and restoration. The patient was completely satisfied with the treatment, and the tooth remained asymptomatic for more than 8 years, until a subgingival fracture occurred (Figure 1C). Clinical and radiographic examination revealed a deep subgingival fracture with limited remaining tooth structure. A treatment plan was formulated for extraction, and informed consent was obtained from the patient.

## 3. Laboratory Examination and Analysis

For permission to study the extracted tooth, approval was obtained from the Institutional Review Board (IRB) of Seoul National University Dental Hospital, Seoul, Korea. Following extraction, the tooth was embedded in an acrylic block and the apical root segment (5 mm) trimmed with a slow-speed, water-cooled diamond saw (Isomet Low Speed Saw; Buehler, Lake Bluff, IL, USA). This root segment was split longitudinally, washed briefly in distilled water, and sputter coated with platinum for scanning electron microscopy (SEM; S-4700, 15 kV; Hitachi, Tokyo, Japan). The interface between the obturation material in the root canal and the surrounding root dentin were carefully examined and the intratubular microstructure was observed. Elemental composition of intratubular mineralized precipitates were analyzed by using energy dispersive spectroscopy (EDS; 7200-H, Horiba, Northampton, UK) to provide qualitative and semi-quantitative measurements of atomic calcium and phosphorous to calculate the superficial calcium-to-phosphorous atomic ratios (Ca/P).

## 4. Results and Discussion

**Figure 2:**
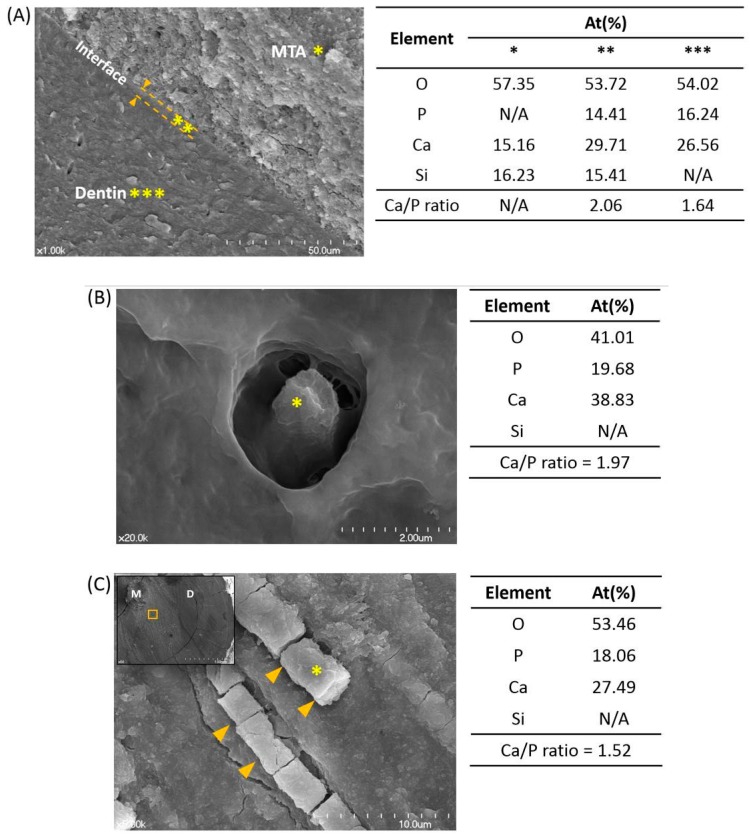
Scanning electron microscopic images that are representative of the root canal dentin and mineral trioxide aggregate (MTA) interface. (**A**) There was an intermediate layer (**) at the interface of MTA (*) and dentin (***) (×1000). (**B**) A dentinal tubule that was occluded by a mineralized structure (×20,000). (**C**) Higher magnification image (×50,000) of yellow boxed area in upper left image showing horizontal cross-sectional view of the root (×50). Biomineralized dentinal tubules (arrowheads) at 1.0–2.0 mm distance from root canal. M: mineral trioxide aggregate; D: dentin. Tables show semiquantitative chemical composition showing Ca/P ratio of the pointed areas (*) in each figure.

This case report provides the very first in vivo-based findings on the clinical effectiveness of MTA as an orthograde root canal filling material. This mandibular canine had developed with chronic (asymptomatic) apical periodontitis, due to the failure of a prior root canal treatment with conventional materials. When the canal was retreated and filled with OrthoMTA, and the tooth properly restored, it remained asymptomatic and fully functional for more than 8 years. Ultimately, when it succumbed to fracture, the roots were carefully extracted and analyzed. Careful microscopic and chemical analyses of the OrthoMTA-filled root canal revealed that there had been a tight seal between the material and surrounding dentin, and there appeared to have been biomineralization within the dentinal tubules (Figure 2). 

OrthoMTA is mainly composed of tricalcium silicate, and contains less heavy metal than the original ProRoot MTA (Dentsply Maillefer) [10]. Previous in vitro studies reported on its use as a root canal filling material, and showed intracanal mineralization at the interface of root canal dentin and OrthoMTA [8,9,11]. Now, this case report presents the first in vivo data which suggest that bioceramic materials are effective for root canal obturation material. They bond to the dentin surfaces, and they promote biomineralization within dentinal tubules. 

Bioceramics can bond to dentin by a process of alkaline etching (whereby the alkaline cement forms a mineral infiltration zone at the interface with adjacent dentin [12]). Accordingly, the root canal obturation material was found to be very closely adapted to the dentin surfaces, as reported in previous studies (Figure 2A) [4,5,6,10]. Furthermore, there was a very thin intermediate layer between the MTA and dentin, where Ca, P, and Si were detected. This suggests that there had been biomineralization on the surface of the material following its hydration, and that there was chemical bonding between the two interfaces. Such structures may have facilitated adaptation of the material onto root dentin, and ensured a tight seal at their interface.

SEM analysis clearly showed that the orifices of dentinal tubules orifices were occluded with mineralized structures. These had Ca/P ratios that were higher than 1.67, probably formed from the precursor-precipitation of the material (Figure 2B) [5,6]. Then, extending in from the orifices and passing along the tubules were intratubular mineralized structures, which had Ca/P ratios ranging from 1.30 to 2.12 (Figure 2C). These were most probably amorphous calcium phosphate precipitates, which formed gradually from calcium ions that leached from MTA and phosphorous ions that were in dentinal fluid [13,14]. Such a process of intratubular mineralization could physically entomb microorganisms [8,15] and deplete intratubular phosphorus ions that are essential for *E. faecalis* survival [16]. Additional XRD or infrared spectrum analyses could have determined crystallinity and phase for the mineralized structures. However, a sample thickness of less than 5 µm limited these analyses to the SEM/EDS that were performed.

These intratubular mineralized structures had Ca/P ratios that appeared to decrease progressively through the dentinal tubule. At the orifice opening that was adjacent to the MTA material, Ca/P ratios were relatively high. Deeper within the tubules, where calcium ions from MTA had further to diffuse, the Ca/P ratios were lower. The biomineralized structures that were around the orifice openings may have promoted a slightly alkaline microenvironment that would have reduced inflammation. However, those with lower Ca/P ratios that were found deep inside tubules may have promoted an acidic microenvironment that could provoke a phase transition of calcium phosphate into unstable forms that include carbonates [17,18]. Such carbonates could interact with fibronectin in the dentin matrix and reduce permeability [19,20], but they could also allow the microleakage of bacterial virulence factors and a recurrence of periapical pathosis.

Nevertheless, in this case the intraoral clinical radiographs revealed that there had been periapical healing and complete resolution of the radiolucency following retreatment with MTA. The radiographs also showed that there was an acceptable density for the root canal filling with MTA. Additional techniques would have been necessary to identify the presence of voids and determine their extent. Such internal voids macro- and microporous structures within the MTA fillings have been reported to be directly related to the leakage of the root filling [21]. The shortcomings are due to the difficulty of delivering MTA through an orthograde approach. Additionally, there may be inadequate powder/water ratios, insufficient packing, or excessive water evaporation that are all associated with an inferior quality of orthograde MTA [21]. 

However, despite the presence of such porous structures within MTA fillings and the difficulty of their placement, their capacity to induce intratubular biomineralization may be particularly beneficial. It is speculated that calcium ions originate from the material in the hydroxide form, and in a highly alkaline environment, they induce mineralization on both the surface of the material, as well as within the dentinal tubules. 

This case report has clearly documented intratubular biomineralization by the careful SEM examination and EDS analysis of a root canal that had been filled with MTA for more than 8 years. Leaving many other issues aside, this report partly shows MTA as a root canal filling material in terms of long-term stable intratubular biomineralization. Further in-depth investigations will be required to determine the crystallinity and the phase of biomineralized structures that were found. Such bioactive properties would support the use of MTA as a root canal filling material, despite the irretrievability of the material for retreatments.
